# Evaluation of the Cancer Nurses Society of Australia mentorship program for cancer nurse practitioners: brief research report

**DOI:** 10.3389/fmed.2026.1753935

**Published:** 2026-07-22

**Authors:** Gillian Kruss, Victoria Team, Vanessa Clothier, Ladan Yeganeh, Suzanne Bartlett, Gillian Blanchard, Rebecca Booth, Michael Cooney, Michael Fitzgerald, Justin Hargreaves, Kristin Linke, Vicki McLeod, Marisa Stevens, Julia Morphet, Olivia Cook

**Affiliations:** 1 Monash Breast Services, Monash Health, Moorabbin Hospital, East Bentleigh, VIC, Australia; 2 Monash Nursing and Midwifery, Faculty of Medicine, Nursing and Health Sciences, Monash University, Clayton, VIC, Australia; 3 Dandenong Emergency Department, Dandenong Hospital, Monash Health, Dandenong, VIC, Australia; 4 Department of Medical Oncology, Paula Fox Cancer and Melanoma Centre, Alfred Health, Prahran, VIC, Australia; 5 Department of Medical Oncology, Calvary Mater Newcastle, Waratah, NSW, Australia; 6 Hunter Valley Oncology, Maitland Cancer Centre, East Maitland, NSW, Australia; 7 Chris O’Brien Lifehouse, Camperdown, NSW, Australia; 8 Cancer Services, Northern Hospital, Northern Health, Epping, VIC, Australia; 9 Department of Medical Oncology, Flinders Medical Centre, Bedford Park, SA, Australia; 10 Department of Medical Oncology, Bendigo Health, Bendigo, VIC, Australia; 11 Department of Medical Oncology, The Queen Elizabeth Hospital, Woodville South, SA, Australia; 12 Department of Medical Oncology, Monash Health, Moorabbin Hospital, East Bentleigh, VIC, Australia; 13 Department of Oncology, St Vincent’s Private Hospital, East Melbourne, VIC, Australia

**Keywords:** health professional education, hematology, mentorship program, nurse practitioner, oncology, palliative care, self-assessment

## Abstract

**Introduction:**

Cancer nurse practitioners (CNPs) provide specialized care for patients with complex cancer needs. CNPs undertake advanced assessment, diagnosis, prescribing, and management of treatment-related toxicities and end-of-life care. Beyond clinical care, Nurse Practitioners (NPs) engage in leadership, education, and research. However, many student and novice CNPs report challenges developing confidence and capability across all domains. Structured mentorship may support this transition.

**Methods:**

A one-year mentorship program matched novice/student CNPs with experienced CNPs. As part of this program, a self-assessment instrument—the Nurse Practitioner Self-Assessment Tool (NP-SAT)—was developed, validated and piloted to measure participants’ knowledge, skills, and confidence. A mixed-methods design incorporated surveys and semi-structured interviews with student and newly endorsed CNPs (*N* = 10) and their mentors (*N* = 10) to identify perceived needs and elicit enablers and barriers to their role transition.

**Results:**

Participants worked in metropolitan (50%) and regional/rural (50%) settings, with most (80%) in public healthcare. Post-program, there were statistically significant increases in clinical knowledge and research domains. Participants were satisfied and found the program feasible.

**Conclusion:**

This study is the first to develop and implement a cancer nurse practitioner mentorship program. The validated tool may inform future CNP workforce and education strategies and be adapted for other NP specialties. Despite reported positive outcomes, and good feasibility and acceptability, due to the limited sample size and absence of a control group, the findings regarding the program’s effectiveness should be regarded as preliminary.

## 1 Introduction

In Australia, nurse practitioners (NPs) are highly experienced, Master’s-qualified advanced practice nurses endorsed by the Nursing and Midwifery Board of Australia to independently diagnose and manage a variety of acute and chronic health conditions across the lifespan. NPs working in medical oncology, radiation oncology, surgical oncology, hematology and/or palliative care specialty areas and managing patients with highly complex needs are referred as Cancer Nurse Practitioners (CNPs). These patients frequently benefit from CNPs’ clinical expertise and supportive care as they promptly triage, manage their patients’ symptoms of disease and/or side effects of treatments, and provide educational information to consumers (1, 2). CNPs manage complete episodes of patient care including: advanced clinical assessment, ordering and interpreting diagnostic tests, formulating a diagnosis, prescribing medications, supporting and educating patients, and managing their treatment related toxicities and/or end of life care (2, 3). Their role can improve patient outcomes, quality of life and satisfaction with care by providing timely access to specialized health care and enabling early interventions that can prevent rapid clinical deterioration, and reduce hospital admissions (3–5).

Along with an extended scope of clinical practice, it is an expectation that all Nurse Practitioners (NPs) also engage in three other domains of practice—leadership, education and research (6). To assist with the longevity and sustainability of new NP roles it is essential that student and novice NPs are provided individualized support and guidance. One way in which this could be achieved is through mentorship (7, 8). Mentorship for NPs is particularly beneficial for growing new NP roles, as it can provide opportunities for improved role transition, job satisfaction and succession planning (5, 9–12). However, the transition of student and novice NPs within the oncology specialty has had minimal research to date both in Australia and globally.

Recognizing this need and in keeping with their strategic plan, the Cancer Nurses Society of Australia (CNSA) Cancer Nurse Practitioner Specialist Practice Network (CNP SPN) committee designed a new one-year pilot mentorship program for their members. Ten mentors and ten mentees were paired using a process of practice field specialty concordance. This process involved the committee members of CNSAs CNP SPN matching the participants who work in the same specialty cancer area. Mentees that expressed their interest in participation were recruited for this project. A power analysis was not conducted, as the sample size was determined by the pilot nature of the study and the availability of funding. Both mentors and mentees attended a virtual workshop via Zoom™ (13) facilitated by the committee members to meet each other and learn about the mentorship program. The content discussed at this workshop included an explanation of: (1) mentoring including the rationale, aims and benefits of a mentorship program; (2) the design and structure of the CNP mentorship program; and (3) the design and methodology of the research. The participants were also given the links to the following recommended resources at the workshop: the Health Education and Training (HETI) Guide for Mentees^1^ and the Australian College of Nurse Practitioners (ACNP) shared plan and agreement.^2^ The mentors and mentees were asked to meet monthly for 12 months via teleconferencing (platform of their choice) for one-hour mentorship sessions. They were also asked to use the results of the mentee’s self-assessment tool to help the mentee set goals and review the progress of the goals throughout the year and at the last mentorship session. This tool was underpinned by Benner’s model (14) thereby allowing participants to rate their performance from ‘novice’ to ‘expert’. Mentees were asked to complete all session reports and email them to the research team. While the report templates included predefined discussion topics, mentees were encouraged to prioritize areas most relevant to their individual learning needs. In their session reports, mentees were asked to select the main themes they discussed with their mentors, including (1) case presentation, (2) clinical (prescribing diagnostic, assessment), (3) referral, (4) billing/ the Medicare Benefits Schedule funding/administration support, (5) other domains of practice (leadership, research, education), (6) clinical supervision in the workplace, (7) nurse practitioner model of care, (8) professional issues, and (9) other themes (please specify).

This program aimed to enable more experienced CNP members to mentor, develop, guide and support their student and novice (endorsed as a NP for less than 2 years) members with an intent to increase their knowledge, skills and confidence when transitioning into CNP roles and meet the NP standards for practice (6). ‘Novice’ is the first level of proficiency in the process of skill acquisition in the Dreyfus and Dreyfus Model of Skill Acquisition (15), which included five levels of proficiency: novice, advanced beginner, competent, proficient and expert. Applying Dreyfus and Dreyfus Model of Skill Acquisition (15) to nursing practice, Benner (14) described ‘novice’ health professionals as ‘beginners’, who have ‘no experience with the situations in which they are expected to perform tasks’. Benner (14) characterized them as facing ‘the inability to use discretionary judgment’ and using ‘context-free rules to guide their task performance’. In this project, we considered ‘novice NPs’ as NPs endorsed for autonomous practice by Australian Health Practitioner Regulation Agency (AHPRA) for <2 years and nurses undertaking their Masters of Nursing Practice course that were not yet endorsed. This manuscript comprises a brief report of the CNSA mentorship program for novice CNPs implementation and evaluation.

## 2 Methods

### 2.1 Project aims

The overarching project aim was to develop, implement and evaluate a 12-month pilot mentorship program for student and novice CNPs. The specific objectives of the program were to:

1. Develop a self-administered tool that assesses the knowledge, skills and confidence of student and novice NPs and test the content validity of this tool.
2. Investigate the impact of a pilot mentorship program on the self-assessed knowledge, skills and confidence of student and novice CNPs in relation to NP domains of practice.
3. Explore the feasibility and acceptability of a pilot mentorship program from the perspective of mentors and mentees.
4. Explore the experiences and perceptions of student and novice CNPs transitioning to CNP roles.
5. Explore the experiences of mentees and mentors participating in a pilot CNP mentorship program.

The project consisted of four phases.

### 2.2 Phase 1: tool development and content validity assessment

An expert working group comprising CNPs, researchers and educators, developed and validated the Nurse Practitioner Self-Assessment Tool (NP-SAT) (Supplementary File S1). The NP-SAT was designed using the validated Australian Advanced Practice Nursing Self-Appraisal (AAPNSA) Tool (16) as a template, however some of the activities under each of the four domains of nursing practice were modified to include specific NP activities from the Nursing Midwifery Board Australia’s (NMBA) NP Standards for Practice 2021 document (6). The NP-SAT also used Benner’s ‘novice’ to ‘expert’ model (14) to replace the scale in the AAPNSA tool to guide those who use the NP-SAT to rate their own levels of knowledge, skills and confidence in each of these NP activities.

The content validity of the NP-SAT was tested utilizing the Content Validation Index (CVI) methodology (17). The CVI requires expert reviewers to rate the relevance of each item on a scale of 1 = not relevant, 2 = somewhat relevant, 3 = quite relevant, 4 = highly relevant, and the clarity of each item on a 4-point scale of 1 = not clear, 2 = somewhat clear, 3 = quite clear, 4 = highly clear. The CVI was calculated to quantitatively assess the content validity of multi-item scales. A CVI for each item (I-CVI) on the NP-SAT tool was calculated by dividing the number of experts ranking the item 3 or 4, by the number of experts. Then the average CVI across items was computed to achieve the CVI for the total instrument. Items that scored an instrument CVI of 0.78–0.8 or greater indicated good content validity. Any items with an I-CVI less than 0.78 were reviewed and any items with very low I-CVI were deleted (17).

### 2.3 Phase 2: survey to assess mentees’ knowledge, skills and confidence levels

We adopted a quasi-experimental pre/post-test study design. Following the informed consent, mentees were emailed the link to the pre-program baseline questionnaire NP-SAT. Immediately after completing the program, mentees were sent an email and asked to complete the post-test NP-SAT online. Categorical variables were reported as number (percentages) and continuous variables were expressed as median/interquartile range (IQR) or mean/standard deviation (SD). Wilcoxon signed-rank test was used to compare the total scores in each domain from pre- and post-program. Effect size (ES) Cohen’s d was calculated to measure the magnitude of changes in scores between baseline and each time point using the sample SD of the mean difference. A *p* value <0.05 was considered statistically significant.

### 2.4 Phase 3: survey to assess the feasibility and acceptability of the program

To evaluate the feasibility and acceptability of the mentorship program, mentors and mentees were asked to complete a brief survey after completing the program. It comprised 30 items, each rated on a 5-point Likert scale: from ‘strongly disagree’ to ‘strongly agree’. The items covered a comprehensive range of domains, including program structure and logistics, organizational support, technical accessibility, preparation and guidance, interpersonal relationships, program outcomes and impact, and administrative support.

### 2.5 Phase 4: interviews to explore mentees’ and mentors’ perceptions/experiences

Semi-structured individual interviews of the mentees (*N* = 10) were conducted by OC pre-program to explore their experiences and perceptions of the mentorship program. Conducting the pre-program interviews, we aimed to identify mentees’ perceptions of transitioning to new nurse practitioner roles, their needs, decision to participate in the program, and program expectations. Both the mentees (*N* = 10) and the mentors (*N* = 10) were interviewed by VT at the end of the mentorship program to explore their experiences of and perceptions regarding the feasibility and acceptability of the program. These interviews were conducted online via Zoom™ (13). Transcripts of the Zoom™ recordings were imported into NVivo 14™ and coded by VT, using a coding framework developed in consultation with the research team. Deductive content analysis (18, 19) was undertaken whereby data were coded to the domains of practice of the NMBA NP Standards for Practice, including the clinical, research, education and leadership domains (6). We used a three-level coding approach. The lowest level involved the identification of program-specific constructs, namely the needs discussed by CNPs and their mentors. These program-specific constructs were then grouped into CNP roles and role-related activities, forming the second level of coding. These roles were subsequently clustered into overarching themes aligned with domains derived from the NMBA NP Standards for Practice. The developed coding framework was reviewed by the research team, who suggested refinements to the lower-level codes. The coder (VT) regularly consulted with the research team during scheduled meetings throughout the coding process and incorporated their feedback accordingly.

## 3 Results

### 3.1 Phase 1: the NP-SAT development

Six Australian expert NPs from different specialties, including cardiology, primary health care, clinical oncology, gerontology and chronic diseases, reviewed the content of the tool. Three rounds of reviews were conducted before final consensus on the relevance and clarity of each item listed in the NP-SAT was reached. All revisions made in NP-SAT are detailed in Table 1. Following the NP-SAT revisions, the final average I-CVI and S-CVI were calculated, and the results are shown in Table 2. The final tool contained 44 items with the S-CVI of 0.92 for relevance and 0.95 for clarity and was used by the mentees in the CNSA CNP SPN mentorship program to assess their baseline and post-program levels of knowledge, skills and confidence for each item in the tool.

**Table 1:** Revisions of NP-SAT following the experts reviews.

Domain	Original item	I-CVI	Changes (after 1st review)	I-CVI	Changes (after 2nd review)	I-CVI
Optimizing Health Systems	3.2. Consults with others regarding conduct of projects or presentations	I-CVI < 0.83-relevance	Item removed	–	–	–
Optimizing Health Systems	3.3. Contributes to, consults or collaborates with other health care personnel on recruitment and retention activities	I-CVI < 0.83-relevance	Item removed	–	–	–
Education	4.6. Facilitates professional development of nursing staff through education	I-CVI < 0.83-clarity	Item removed (The role of the NP in education is better represented by other items)	–	–	–
Research	5.1. Leads or contributes to clinical research	I-CVI < 0.83-relevance	Item re-worded to ‘Leads or contributes to research within capacity’	I-CVI < 0.83-Relevance and clarity	Items 5.1 and 5.2 were combined and reworded to ‘Leads, contributes to or uses research and quality-improvement programs to guide and develop practice/s’	I-CVI = 0.83 for relevance and clarity
Research	5.2. Actively participates in assessment, development, implementation and evaluation of quality-improvement programs	I-CVI < 0.83-relevance	Item re-worded to ‘Contributes to the assessment, development, implementation and evaluation of quality-improvement programs within capacity’	I-CVI < 0.83-Relevance and clarity		
Research	5.4. Identifies funding sources for the development and implementation of clinical projects/programs	I-CVI < 0.83-relevance	Item removed	–	–	–

**Table 2:** The content validity results of the Nurse Practitioner Self-Assessment Tool (NP-SAT).

Subscale	Number of items in the original tool	Number of valid items in the final tool	Average I-CVI–Relevance	Average I-CVI–Clarity
Clinical care	18	18	0.96	0.95
Optimizing health systems	9	7	0.88	0.95
Education	7	6	0.97	0.94
Research	7	5	0.83	0.97
Leadership	8	8	0.89	0.92

### 3.2 Phase 2: mentees’ knowledge, skills and confidence levels

#### 3.2.1 Sample description and demographics

Ten participants completed the NP-SAT pre- and post-program. All participants held a Masters level degree, and most were endorsed NPs (*n* = 9, 90%). Half of the sample (*n* = 5, 50%) worked in metropolitan settings, with four people working regionally and one rurally. Most (*n* = 8, 80%) worked in the public health system, and two worked in the private system. Most (*n* = 7, 70%) reported that they worked full-time. They worked in a combination of practice settings, including medical oncology (*n* = 9, 90%), hematology (*n* = 3, 30%), radiation oncology (*n* = 2, 20%), and palliative care (*n* = 1, 10%). (Note that some participants reported working in multiple settings, so this sums to >10). The participants worked in in-patient (*n* = 2, 20%) and out-patient settings (*n* = 3, 30%), with half of the participants reporting they worked in both in-patient and out-patient settings (*n* = 5, 50%). Half of the sample indicated they were endorsed as an NP but were not working as an NP (*n* = 5, 50%). The remaining sample were inexperienced NPs, with one not yet endorsed, three having worked as an NP for 4–7 months, and one having 23 months of experience.

#### 3.2.2 Comparison of pre- and post-test scores

Data were not normally distributed, so Wilcoxon signed-rank test was used to compare the total scores in each domain from pre- and post-program. Wilcoxon signed rank test was used to compare scores from repeated measures at two points in time, to assess whether their mean ranks differ. The test was used to determine any statistical difference in median group score pre and post. Because the Kolmogorov–Smirnov test has low power in small samples, histograms were used to identify the skew of the data. Histograms were used to visually assess the distribution of the data and identify its overall shape, spread, and skewness. In addition, both skewness and kurtosis values were largely negative, indicating that the distribution deviated from normality and was relatively flat, indicating that parametric assumptions were not met.

#### 3.2.3 Clinical knowledge domain

The clinical knowledge domain was the domain which demonstrated the greatest score increase following the NP program. The median score for the Clinical knowledge domain was 56.5 pre-NP mentoring, and 68 post-NP mentoring (from a potential total of 90). The median increase of 11.5 was statistically significant (*p* = 0.028), with a large effect size (*r* = 0.49, *z* = −2.194).

#### 3.2.4 Optimizing health systems domain

The median score for the domain Optimizing health systems domain was 22.5 pre-NP mentoring, and 27 post-NP mentoring (from a potential total of 35). The median increase of 4.5 was not statistically significant (*p* = 0.4).

#### 3.2.5 Education domain

The median score for the Education domain was 23 pre-NP mentoring, and 25 post-NP mentoring (from a potential total of 30). The median increase of 2 was not statistically significant (*p* = 0.212).

#### 3.2.6 Research domain

The median score for the Research domain was 13 pre-NP mentoring, and 15.5 post-NP mentoring (from a potential total of 25). The median increase of 2.5 was statistically significant (*p* = 0.037), with a large effect size (*r* = 0.47, *z* = −2.088).

#### 3.2.7 Leadership domain

The median score for the Leadership domain was 22.5 pre-NP mentoring, and 25.5 post-NP mentoring (from a potential total of 40). The median increase of 3 was not statistically significant (*p* = 0.184).

### 3.3 Phase 3: feasibility and acceptability of the program

#### 3.3.1 Demographic data

The demographic data for the mentees is the same as reported in Phase 2.

#### 3.3.2 Satisfaction and feasibility

Responses to the satisfaction and feasibility questions were overwhelmingly positive, with most participants selecting either ‘agree’ or ‘strongly agree.’ The few deviations from these ratings were primarily related to challenges in coordinating mutually agreeable times for monthly mentorship sessions, as reflected in responses to the item “It was easy to coordinate mutually agreeable times each month.” Some participants were neutral in their response regarding whether their workplace was supportive of mentoring occurring during work hours. One mentee felt instructions on what to expect from the mentorship program were not clear. Four mentees were neutral on whether this process helped them with setting goals. Two mentees disagreed that the program helped with research.

### 3.4 Phase 4: pre- and post-program perceptions and experiences

Mentees reported on the topics covered during mentorship sessions with a summary presented in Table 3. The most frequently discussed topics during mentorship sessions were professional issues, clinical case presentation, assessment and prescribing, NP Model of care, and leadership. The less frequently discussed topics were billing, Medicare Benefits Schedule funding, administrative issues, and referrals.

**Table 3:** Summary of mentorship sessions.

Dyad (*N* = 10)	Number of sessions	Shortest session (min)	Longest session (min)	Mean session duration (min)	Most frequently discussed themes
Dyad 1	11	30	45	41	Professional issues, clinical assessment, clinical prescribing, leadership
Dyad 2	10	30	45	33	Professional issues
Dyad 3	10	20	30	28	Professional issues, clinical assessment, clinical prescribing, leadership
Dyad 4	12	15	45	27	Professional issues, clinical assessment, clinical diagnostic, NP model of care
Dyad 5	12	60	75	61	Clinical assessment, clinical diagnostic, professional issues
Dyad 6	12	30	60	33	NP model of care
Dyad 7	12	60	90	63	Clinical prescribing, case presentation, clinical diagnostic, NP model of care
Dyad 8	10	20	50	37	Professional issues, clinical prescribing, clinical diagnostic, clinical supervision in the workplace
Dyad 9	10	30	60	54	Clinical assessment, research, professional issues
Dyad 10	10	45	90	62	Clinical prescribing, leadership, clinical supervision in the workplace, NP model of care

#### 3.4.1 Expressed needs and program expectations

During pre-program interviews, mentees discussed key motivators for joining the program such as developing goals; learning mentor’s experience of transition to their NP role; receiving guidance and support; and reducing professional isolation. Within the clinical domain, mentees were hoping to receive guidance on transitioning to a more advanced scope of clinical practice for an NP and aspired to improve their skills through independently assessing and managing their patients’ symptoms and side effects of treatment. Within the research domain, a predominant pattern demonstrated by the mentees was not knowing where and how to get started, recognizing their scant research experience. Within the education domain, mentees noted they felt passionate about educating patients and other staff and knowing how to enrich their own learning. Within the leadership domain, mentees reported improved confidence, an expected outcome of the mentorship program. This expectation not only related to their self-confidence as a safe, autonomous practitioner but also earning the confidence and trust of their medical colleagues. Mentees also recognized there was a need to educate other health professionals about their role and establish and maintain good communication with other departments (Supplementary Table S2).

#### 3.4.2 Program participation experiences

Mentoring helped mentees transition to working to an expanded scope of practice particularly in the clinical domain of their practice. Within the clinical domain, mentees acknowledged mentors’ support helped to improve their advanced assessment skills, clinical decision making, diagnostic reasoning and prescribing practices (Supplementary Table S3). Mentees benefitted from their mentors’ guidance on how to safely prescribe medicines and monitor their effectiveness; interpret the Pharmaceutical Benefits Scheme and other guidelines to inform their prescribing practices; and learn processes for prescribing specific medications, such as opioids, steroids or antibiotics. Unique topics related to the oncology specialty area were identified such as discussion points on immunotherapy toxicities, symptom management and adjusting chemotherapy doses for abnormal blood results. Mentees’ research goals were not being set due to competing interests in the clinical domain. Research that was considered centered around the evaluation of new NP roles being embedded into the health service. Mentors generally acknowledged research with their mentees, and how important it is to the NP role. Within the education domain, the topics discussed were concentrated on patient education and education initiatives for peers. Furthermore, succession planning for new cohorts of NP students and the future mentoring role the current NP (mentee) may have in those new recruits’ careers was frequently discussed. Within the leadership domain, mentees acknowledged improved self-confidence as an autonomous practitioner. Mentees valued mentor’s advice on how to communicate to other health professionals about their role and to establish communication with other departments.

## 4 Discussion

### 4.1 Mentorship as a catalyst for standards-aligned advanced practice

This four-phase study has evaluated a 12-month pilot mentorship program for novice CNPs, the first of its kind in Australia. The primary focus for novice CNPs, as they transition to more advanced scopes of practice, appears to align predominantly with the clinical domain of the NP Standards for Practice (6). Our findings indicate that research, education, and leadership domains hold significantly lower priority for novice CNPs compared to clinical practice. Perhaps this is unsurprising given the need for novice NPs to focus on their extended scope of practice and prescribing to ensure that they are practicing safely in their new role. The Nurse Practitioner Standards for Practice (6) scaffold above the Registered Nurse Standards for Practice (20). The curriculum for both concentrates on the clinical attributes of the emerging clinician.

Adapting to working autonomously may be stressful for novice CNPs. They reported a shift from feeling confident, connected, and capable working in a role with less responsibility, to experiencing an initial lack of confidence and feelings of isolation when beginning a NP role that demanded more accountability, clinical decision-making, and less supervision. These experiences align with the findings of previously published studies (21–23). Moreover, the novice NPs reported a perceived lack of confidence in themselves, or from their peers/colleagues within their new advanced scope of clinical practice. Doubting their clinical decision-making skills led to NPs taking extra time to make clinical decisions, thus further adding to their stress levels as they faced more pressure to complete their required workloads. MacLellan and co-authors (22) concur noting that consequences of NP transition include a loss of identity, loss of confidence, marginalization and isolation. In literature, this role transition period is also called the NPs’ role socialization (24), with studies (25, 26) reporting that the recently graduated NPs may be inadequately socialized into their role. Moreover, some researchers (27) also highlight that the recent NP graduates perceive themselves unprepared for their NP role regardless of their previous experience working as a Registered Nurse (RN); and previously experienced specialist nurses feeling like novices in their NP role (28). Mentorship is regarded as the main enabler to the role socialization (29, 30). The value of mentor support during the time of transition is significant, providing essential guidance, confidence building, and professional development for mentees (31–33).

The mentorship program enhanced mentees’ confidence in expanding their clinical scope and improved their clinical decision-making. This finding is consistent with the work by Reabold and Quattrini (5) who also note that participating in an NP mentorship program empowers NPs to develop their clinical and professional skills. Their work further shows that mentorship programs facilitate knowledge sharing, which supports NP development and ultimately enhances the quality of care. Presenting oncology case studies during mentorship sessions promoted critical thinking and clinical reasoning (34). Our model, which paired mentees and mentors from the same clinical specialty, fostered nuanced discussions and shared lived experiences—helping novice CNPs feel understood. This type of pairing contrasts with other models that deliberately matched mentors from different specialties to focus on professional rather than specialty-aligned clinical issues (35). Previous studies (36–38) have shown that experiential learning approaches that were similar to those used in our mentorship model effectively support NPs in practicing to their full scope and advancing professionally. While participants in this program did not explicitly report discussing research during their mentoring sessions, we found a statistically significant improvement in their self-reported research domain scores. This finding is consistent with previous reports indicating that mentored NPs were more to be engaged in academic activity or hold an academic rank than those who were not (39).

Mentoring was particularly valuable in supporting prescribing practices. Given the variation in protocols and legislation across Australian states and territories, mentees benefited from the opportunity to clarify prescribing requirements specific to their practice context and location through discussions with their mentors. While CNPs commenced their role with foundational pharmacodynamic and pharmacokinetic knowledge from their university coursework, mentors played a critical role in bridging the gap between theoretical understanding and practical application. Prescribing regulations for NPs vary internationally and across Australian states, complicating mentorship in oncology (39). While the Nursing and Midwifery Board of Australia provides standardized prescribing guidelines (40), state-specific prescribing legislation varies. These inconsistencies can challenge cross-jurisdictional mentorships, potentially leading to conflicting guidance. Aligning mentors and mentees within the same jurisdiction would enable shared understanding of regulations relating to NP roles and more effective support.

Mentors played a crucial role in assisting mentees in either revising or developing a model of care tailored to their organization. Many mentors had direct experience in establishing their own models of care and were able to provide valuable insights by sharing their experiences and existing frameworks with novice CNPs. Mentors helped mentees adapt and implement effective models of care in their respective workplaces, aligning with outcomes reported in other programs (41, 42).

Novice NPs reported feeling confident with their educational skills and the ability to scaffold their existing knowledge, skills, and experiences with patients, and other peers or health professionals. They identified the need to use education as a strategy for succession planning and growing their NP service by educating, mentoring, and inspiring other nurses to become NPs, which is congruent with other Australian studies (43) on NPs roles in other healthcare fields.

Novice CNPs expressed interest in participating in research, however their motivation was largely driven by external pressures—such as the need to demonstrate the value of their role, meet managerial targets, and support business cases for new CNP positions. Despite this interest, research activities were often deprioritized as clinical and leadership responsibilities took precedence. While NPs express a strong interest in contributing meaningfully to research, many face uncertainties about how to begin and how to navigate involvement beyond meeting ongoing practice requirements, such as integrating evidence into clinical care. Time constraints and limited resources further complicate their ability to initiate or participate in research activities (44). To address these challenges, it is essential for health services to develop clear, supportive strategies that actively engage NPs in research. These strategies may include creating accessible pathways for participation, offering mentorship and training opportunities, and embedding research into clinical roles in ways that are feasible within busy practice environments. By doing so, organizations can empower NPs to contribute to research in impactful ways that enhance both professional development and patient care.

A significant challenge reported by mentees was the transition from Registered Nurse to CNP, which required them to assert leadership and clearly communicate their evolving responsibilities to colleagues, also reported by van Kraaij et al. (45). Many found it difficult to explain to nursing peers that they could no longer perform some RN duties alongside their extra CNP responsibilities. Mentors, having faced similar challenges, were able to empathize and offer practical guidance. Their lived experiences provided mentees with strategies for navigating these conversations, helping them build confidence in asserting their new roles within the healthcare team, as also reported by Triglianos and associates (46).

### 4.2 Strength and limitations

This study addresses an important issue in nursing workforce development, supporting novice NPs through structured mentorship programs specifically for NPs in the oncology settings, where clinical complexity is high. It is the first to examine the feasibility, acceptability, and experiences of such a program while exploring the transition experiences of CNPs into their new roles. Previous studies on mentorship programs for NPs support the findings of this research, highlighting that mentoring facilitates smoother role transitions and provides essential support and guidance during this critical period (22, 36–38, 47). This was a structured intervention with mentor–mentee pairings and clearly defined monthly sessions over a 12-month period. This clarity enhances reproducibility and offers a practical framework that other organizations could readily adopt. Clear reporting of mentorship session details, including duration, topics discussed, and frequency, provides useful insight into program implementation. The integration of quantitative (pre/post survey) and qualitative (semi-structured interviews) methods provides a richer understanding of the mentorship program outcomes and participant experiences.

This research was also the first to develop and pilot a new self-assessment tool specifically for NPs titled the Nurse Practitioner Self-Assessment tool (NP-SAT), evaluating knowledge, skills and confidence among novice CNPs. Content validity was assessed using the Content Validity Index (CVI), which strengthens the methodological approach. The conceptualization and development of this tool was based on previous psychometric testing of similar tools which identify the learning needs of cancer nurses called the Cancer Nurse Self-Assessment tools (CaN-SATs) (48, 49). Items in the research domain received the lowest consensus ratings and thus warrant further exploration around NP’s apparent lower prioritization of this domain of practice. The development and psychometric testing of the NP-SAT revealed some limitations, particularly regarding its context-specific applicability beyond the Australian context. The tool was based on the Australian NP Standards for Practice (6) and AAPNSA (16), and validated solely by Australian expert NPs, which confines its relevance to national use and limits international utility. Psychometric testing did not include reliability assessment, such as Cronbach’s alpha or test–retest reliability with a large cohort, reducing the robustness of its validation. Further psychometric testing, including validation by international experts and reliability testing with a larger CNP cohort is recommended to enhance the tool’s applicability and credibility.

Although the sample size was appropriate given the available budget and resources, the insights gained were based on a limited cohort of ten mentees that completed the pre- and post-intervention assessment. While the findings offer valuable preliminary perspectives for future CNP mentorship programs, the small sample size substantially limits statistical power and generalizability of the findings. Given the small sample size, the statistical significance should be interpreted cautiously. To strengthen the evidence base, future research should involve a larger cohort to validate or challenge these initial findings. Moreover, the study uses a pre-post quasi-experimental design without a comparison group, making it difficult to attribute improvements solely to the mentorship program. Reliance on self-reported outcomes, which were measured using self-assessment of knowledge, skills, and confidence may introduce potential bias and may not reflect objective competency improvement of the participating CNPs. Potential selection bias should be considered, as participation in the mentorship program was voluntary, which may have resulted in an overrepresentation of highly motivated CNPs seeking to enhance their knowledge, skills, and confidence. Many aspects of the study are relevant to mentoring CNPs within the Australian context. However, specific themes, perspectives, and experiences may vary significantly across countries with different regulatory frameworks and NP practices (50). Therefore, the design of this pilot study could be adapted to meet the needs of CNPs in other regions, considering country-specific regulations and clinical environments.

## 5 Conclusion

A structured cancer NP mentorship program was considered feasible and acceptable to the participants of this pilot program. The mentorship program supported novice CNPs around obtaining role clarity; working independently and to full scope of practice; and dealing with challenging relationships and stress at work. Furthermore, this pilot project confirmed to the CNSA CNP SPN the merit of continuing their ongoing operation of a mentorship program for its members. The validated NP-SAT survey will also be useful for identifying the learning needs of CNPs and measuring whether future mentorship programs are effective at increasing the knowledge, skills and confidence levels of participating mentees. However, due to the limited sample size and absence of a control group, the findings regarding the program’s effectiveness should be regarded as preliminary.

## Data Availability

The original contributions presented in the study are included in the article/Supplementary material, further inquiries can be directed to the corresponding author.

## Footnotes

1
https://www.heti.nsw.gov.au/__data/assets/pdf_file/0003/428826/The-Learning-Guide-2020.pdf

2
https://www.acnp.org.au/mentoring
