# Supplementary material for: Evaluation of the Cancer Nurses Society of Australia mentorship program for cancer nurse practitioners: brief research report
Source: Front Med (Lausanne). 2026 Jul 22;13:1753935. doi: 10.3389/fmed.2026.1753935 (PMC13449700; doi:10.3389/fmed.2026.1753935)
Supplement: Supplementary file 1 [file Data_Sheet_1.pdf]

Supplementary File 1. The Nurse Practitioner Self- Assessment Tool (NP-SAT)

Nurse practitioner mentorship program-NP-SAT - Version (2)

The Nurse Practitioner Self- Assessment Tool (NP-SAT)

Introduction

This NP-SAT has been created for use by student and endorsed nurse practitioners (NPs) to self-assess their levels of knowledge, skills and confidence in activities critical to the expected domains and advanced scope of clinical practice of a NP. Rather than assessing competency, the aim of this tool is to help NPs identify and address activities or areas of their practice from the 4 domains (clinical, education, research and/or leadership) of the NP Standards for Practice that require further development (Nursing and Midwifery Board of Australia – NMBA, 2021). NPs at any stage of their career can use the results from this tool to develop strategies that will help them address their ongoing learning needs and to continually evolve and expand their scope of practice in any of these domains.

The content and structure of the Australian Advance Practice Nursing Self-Appraisal Tool (the Advance Tool) was chosen to help design the NP-SAT as it has been extensively researched and is a validated tool that is embedded into clinical practice (Gardner et. al. 2017). The Advance Tool comprises five domains of nursing practice expected of an advanced practice nurse (APN) including Clinical Care, Optimizing Health Systems, Education, Research and Leadership, four of which are the same domains from NMBAs' NP standards of practice (Gardner et. al. 2017; NMBA, 2021). As NPs are trained and endorsed by the NMBA to practice at a more advanced nursing level than those required of a registered nurse working as an APN, additional descriptions of activities specific to the NP's scope have been taken from the 2021 NMBA's NP Standards for Practice and added under the relevant domain in the NP-SAT.

The self-assessment component of the Advance Tool has also been modified for the NP-SAT. Rather than using the Likert scale that was used in the Advance Tool to rate the extent in which individual nurses engage in an activity, the Benners model (Benner, 1982) has been incorporated to allow nurses to rate themselves on another Likert scale from novice to expert. This scale remains widely used in nursing assessment and most importantly recognises the influence of context of expertise (Dale et. al. 2013). For example, a palliative care NP working in a hospice may rate themselves as an expert for many of the items on the NP-SAT but rate themselves as a novice for the same items in the context of working in another specialty area such as an oncology day unit.

Instructions for completing the NP-SAT

You will be required to complete a self-assessment of your knowledge, skills and confidence levels for each of the activities listed under the five domains of practice at two time points - before you commence the mentoring program and immediately after completing the mentoring program. This information will initially be used to help you and your mentor to set goals for helping you develop and transition into being a NP that is working towards expanding their scope of practice. This information will also be used to evaluate whether your levels of expertise have progressed after a year of participating in a NP mentoring program. You have the option to expand upon your responses in the NP-SAT and provide justification or specific examples of your current practice to correlate with your self-rated level of expertise for each activity.

Knowledge, skills and confidence- consider each element of practice under the headings of the 5 domains. Check the box on the Likert scale (1-5) below that best represents your current level of knowledge/skills/confidence with that activity/element of practice. The scale relates to Benner's model (1982) from novice to expert as defined below:

1. **Novice** - use rules to guide task performance
2. **Advanced beginner** – acceptable care performance, still need support in the clinical setting
3. **Competent** – able to prioritise care and easily incorporate previous experiences in decision-making
4. **Proficient** - able to prioritise care almost automatically and recognise deviations from a norm
5. **Expert** - possess an intuitive grasp of a situation based upon experience and knowledge gained over time
(Dale et. al. 2013, p.197).

1. **Knowledge, skills and confidence- <u>Clinical domain</u>**.
Check the box on the Likert scale (1-5) below that best represents your current level of knowledge/skills/confidence with that activity/element of practice.

	Novice	Advanced beginner	Competent	Proficient	Expert
1.1 Conducts an advanced and comprehensive health and supportive care assessment and examination	☐	☐	☐	☐	☐
1.2 Uses critical thinking and evidence-based knowledge to identify and address the health care needs of patients and the significant factors that may or are impacting on their health outcomes	☐	☐	☐	☐	☐
1.3 Refers for appropriate screening and/or diagnostic investigations/procedures to inform clinical decision making	☐	☐	☐	☐	☐
1.4 Gathers and interprets assessment data to formulate differential diagnoses and plans of care.	☐	☐	☐	☐	☐
1.5 Demonstrates accountability in considering access, cost and clinical efficacy when planning care, investigations and treatments.	☐	☐	☐	☐	☐
1.6 Performs specialty-specific care, interventions and procedures	☐	☐	☐	☐	☐
1.7 Considers quality use of medicines and therapeutic care to safely prescribe non-pharmacological and pharmacological interventions	☐	☐	☐	☐	☐
1.8 Assesses patient/family response/s to care, therapies and interventions and	☐	☐	☐	☐	☐

	Novice	Advanced beginner	Competent	Proficient	Expert
modifies plans of care based on response/s					
1.9 Works in collaboration with the patient and their family/significant others to develop plans of care and communicates these plans to other relevant health professionals	☐	☐	☐	☐	☐
1.10 Collaborates with other health professionals (including medical health professionals) to make and accept referrals as appropriate and to optimise patient's health status	☐	☐	☐	☐	☐
1.11 Provides counselling and education (to the patient and family) regarding their health and illness, treatment options and goals of care.	☐	☐	☐	☐	☐
1.12 Verifies the suitability of evidence-based treatment options including medicines, in regard to commencing, maintaining/titrating or ceasing interventions in consultation with the person receiving care and when needed, other members of the health care team	☐	☐	☐	☐	☐
1.13 Documents appropriately on patient records	☐	☐	☐	☐	☐
1.14 Serves as a consultant in improving patient care and nursing practice based on expertise in area of specialisation	☐	☐	☐	☐	☐
1.15 Facilitates the process of ethical decision-making in patient care	☐	☐	☐	☐	☐
1.16 Coordinates inter/intra disciplinary plan for care of patients	☐	☐	☐	☐	☐
1.17 Facilitates efficient movement of patient through the healthcare system	☐	☐	☐	☐	☐
1.18 Understands duty of care and current legislative and professional regulations governing NP practice, and practices within these boundaries/parameters	☐	☐	☐	☐	☐
Comments – please provide any further information, comments and/or examples on Clinical domain.					

2. Knowledge, skills and confidence- Optimising Health Systems domain

Check the box on the Likert scale (1-5) below that best represents your current level of knowledge/skills/confidence with that activity/element of practice.

	Novice	Advanced beginner	Competent	Proficient	Expert
2.1 Participates in strategic planning for the health care service, department or hospital	☐	☐	☐	☐	☐
2.2 Provides direction for and participates in unit/service quality improvement programs	☐	☐	☐	☐	☐
2.3. Provides leadership in the development, implementation, and evaluation of standards of practice, policies and procedures	☐	☐	☐	☐	☐
2.4 Uses quality indicators to monitor and measure the effectiveness of strategies, services and interventions to promote safe practice	☐	☐	☐	☐	☐
2.5 Advocates for the role of the NP and expansion of NP services when required to improve access to quality and cost-effective healthcare for all	☐	☐	☐	☐	☐
2.6 Critiques health policies for their implications on the NP role and the populations for whom NP's care for	☐	☐	☐	☐	☐
2.7 Influences health policy and practice through leadership and active participation in workplace and professional organisations	☐	☐	☐	☐	☐
Comments – please provide any further information, comments and/or examples on Optimising Health Systems domain.					

3. Knowledge, skills and confidence- <u>Education domain</u>

Check the box on the Likert scale (1-5) below that best represents your current level of knowledge/skills/confidence with that activity/element of practice**.**

	**Novice**	**Advanced beginner**	**Competent**	**Proficient**	**Expert**
3.1 Provides appropriate patient and family education	☐	☐	☐	☐	☐
3.2 Respects the rights of the person to make informed decisions throughout their health/illness experience or episode, whilst ensuring access to accurate and appropriately interpreted information	☐	☐	☐	☐	☐
3.3. Identifies learning needs of various populations and contributes to development of education programs/resources	☐	☐	☐	☐	☐
3.4 Serves as informal educator to staff while providing direct care activities	☐	☐	☐	☐	☐
3.5 Serves as educator and clinical preceptor/mentor for nursing and medical staff and/or students/other health care professionals	☐	☐	☐	☐	☐
3.6 Evaluates education programs and resources and recommends revision as needed	☐	☐	☐	☐	☐
Comments – please provide any further information, comments and/or examples on Education domain.					

4. Knowledge, skills and confidence- <u>Research domain</u>

Check the box on the Likert scale (1-5) below that best represents your current level of knowledge/skills/confidence with that activity/element of practice.

	Novice	Advanced beginner	Competent	Proficient	Expert
4.1 Leads, contributes to, or uses research and quality-improvement programs to guide and develop practice/s	☐	☐	☐	☐	☐
4.2 Contributes to research that addresses identified gaps in the provision of care and/or services and implements research -based innovations for improving the quality of care and services	☐	☐	☐	☐	☐
4.3. Uses research and integrates theory into practice and recommends changes to policy and/or processes	☐	☐	☐	☐	☐
4.4 Identifies clinical data necessary for inclusion in information systems for nursing research and quality assurance projects	☐	☐	☐	☐	☐
4.5 Collaborates with information specialists in the design of data systems for research and quality assurance projects in nursing	☐	☐	☐	☐	☐
Comments – please provide any further information, comments and/or examples on Research domain.					

5. **Knowledge, skills and confidence- <u>Leadership domain</u>**

Check the box on the Likert scale (1-5) below that best represents your current level of knowledge/skills/confidence with that activity/element of practice.

	**Novice**	**Advanced beginner**	**Competent**	**Proficient**	**Expert**
5.1 Articulates and promotes the NP role in clinical, political and professional contexts s	☐	☐	☐	☐	☐
5.2 Disseminates nursing knowledge through presentation or publication at local, regional, national or international levels	☐	☐	☐	☐	☐
5.3. Serves as a resource or committee member in professional organisation/s	☐	☐	☐	☐	☐
5.4 Serves as a consultant to individuals and groups within the professional/lay communities and other hospitals/institutions	☐	☐	☐	☐	☐
5.5 Participates in clinical supervision and clinical practice review	☐	☐	☐	☐	☐
5.6 Represents nursing in institutional/community forums on the educational needs of populations	☐	☐	☐	☐	☐
5.7 Represents a professional nursing image at institutional and community forums; serves as a spokesperson for nursing and the health facility when interacting with other professionals, patients, families and the public	☐	☐	☐	☐	☐
5.8 Collaborates with other healthcare professionals to provide leadership in shaping public policy on healthcare	☐	☐	☐	☐	☐
Comments – please provide any further information, comments and/or examples on Leadership domain.					

References

Benner, P. (1982). From novice to expert. *American Journal of Nursing, 82*(3), 402-407.

Dale, J. C., Drews, B., Dimmitt, P., Hildebrandt, E., Hittle, K., & Tielsch-Goddard, A. (2013). Novice to expert: the evolution of an advanced practice evaluation tool. *Journal of Pediatric Health Care: Official Publication of National Association of Pediatric Nurse Associates & Practitioners*, *27*(3), 195–201. https://doi.org/10.1016/j.pedhc.2011.12.004

Gardner G., Duffield C., Gardner A., Batch M. (2017). The Australian Advanced Practice Nursing Self-Appraisal Tool. DOI: 10.6084/m9.figshare.4669432

Nursing and Midwifery Board of Australia., 2021. 'Nurse Practitioner Standards for Practice'. *Australian Health Practitioner Regulation Agency*. https://www.nursingmidwiferyboard.gov.au/codes-guidelines-statements/professional-standards/nurse-practitioner-standards-of-practice.aspx
