# Supplementary material for: Evaluation of the Cancer Nurses Society of Australia mentorship program for cancer nurse practitioners: brief research report
Source: Front Med (Lausanne). 2026 Jul 22;13:1753935. doi: 10.3389/fmed.2026.1753935 (PMC13449700; doi:10.3389/fmed.2026.1753935)
Supplement: Supplementary file 2 [file Data_Sheet_2.pdf]

Supplementary Table 2. Pre-program interviews with mentees (N=10). Summary of the domains, constructs and illustrative quotes

**Domain**	Construct	Program-specific construct	Illustrative quotes
**Clinical Domain**	Clinical assessment	Assessing patients within the scope of practice/ assessment capability	And since I started the nurse consultant job, I realised I've missed the freedom of being a care coordinator, and then I realised – and the patient context as well, that's why if I get a little bit more skill, maybe I can become a nurse practitioner, I can go back to the outpatient setting, more to see the patient, assess them and help them [Mentee 06 pre-program].
	Clinical case-presentation	Case studies and case management	That's how I would foresee it [the Program]. Case studies and case management, I guess, are already set up elsewhere. So, it's probably just another sounding board as well. And just that first year of support [Mentee 03 pre-program].
	Diagnostic reasoning	Improving clinical diagnostic reasoning skills	One of the things that I will definitely focus on from a clinic perspective is what other skills I can gain and develop to make a nurse practitioner position more valuable. I think ultimately, that is what the nurse practitioner role is. It's to upskill constantly, so, what other skills can I gain [Mentee 03 pre-program].
	Prescribing	Professional knowledge -Increasing awareness of the prescribing guidelines	I'd like to be able to have a better knowledge, and this is not really a mentorship thing though, around prescribing guidelines. Because I think that's one of the things, as much as you do your two Pharmacology units in your course, until you're actually doing it, you've never actually done it. I don't know if that makes sense. I guess a bit more confidence around the prescribing aspect because that's something that's a huge difference from any other nursing role [Mentee 01 pre-program].
		Medication Safety	I think when you go to that nurse practitioner, it's actually you that then has to assess and then determine what needs to be done, whether it's investigations, medications or something that's non-pharmacological, and obviously there's a lot of responsibility that

			comes with that, prescribing it, and, as you'd know, oncology patients often have multiple comorbidities, so, ensuring that anything that you are advising or prescribing for the treatment of a side effect or a symptom, you then have to make sure that that doesn't have any adverse effects on other medications or comorbidities that the patients have too [Mentee 05 pre-program].
	Model of Care	Developing the model of care	There're two models that – or two areas that I think a nurse practitioner role in oncology would work really well. One is in survivorship. We don't have a survivorship program as such, each individual clinician is just doing their own survivorship as they go along. That is one where I think a nurse practitioner can play a significant role and improve the service, and patients can benefit greatly from that, and that would be a nurse led role, a nurse led clinic. The other area that as a general oncology nurse practitioner is toxicity management [Mentee 10 pre-program].
	Scope of practice	Defining the scope of practice	And I think really what I'm looking for in the Mentorship Program is to help me extend that, my practice within that transition period that I've been given to work out how I can work within particularly the CNC role but working at an NP level just not doing the extra thing that NPs do, I guess [Mentee 01 pre-program].
**Research Domain**	Research involvement	Novice researchers	Another goal is I'd love to be involved in a research project. When I did my Masters, I thought I'd hate research, but then I did pretty well in the subject, I actually quite enjoyed looking at all the different ways you can do research, like the qualitative, quantitative. And I really want to get involved in a research project. However, I just don't know where to start [Mentee 04 pre-program].
	Research outcomes	Publishing an article	One of the things that I had wanted to do, which I hope it's not too ambitious, but there's not much literature around models of care for oncology or cancer nurse practitioners. So, one of the things that I had wanted to do, was maybe once I'm established, maybe write it up as a research article, to help contribute to that. So, that's one of the goals that I have, which would be good to have some support with [Mentee 08 pre-program].

**Education Domain**	Patient education	Setting up a survivorship program	So, we're getting more and more patients but less and less staff. We don't have - we're a regional centre that don't have the facilities of [central location]. So, having a nurse practitioner in there that could do those type of things - we have no survivorship program. I can start a survivorship program. I can - because we're such a small centre, our patients get education on the day of treatment. So, things like that, I can build an education session with them before, let them ask questions and have the time to actually think about it rather than freak out while they're getting their infusion [Mentee 04 pre-program].
	Staff education and mentorship	Oncology-specific clinical education	But I think the goal is to expand our service and try and get - educate our staff to try and get these things…, a lot of stem cell stuff we don't do [Mentee 04 pre-program].
		Debriefing	For example, if there is a case study that I might have, it will be a good idea to debrief. Where are you, how was your day today? Well, this and this happened today and this is what I did. It's to be able to say, "Well, yeah, that's OK. That's what you should've –" you know what I mean? [Audio 143692]
	Professional development/ career planning	Planning graduate education	Education-wise, I'll need to continue to learn, so I'm more than happy for someone to let me know the area I can improve on. I was thinking either doing another grad cert in aged care, which will help me do the geriatric assessment or maybe like a PHD in the geriatric oncology. I feel like there's a lot of unknown, that's one of the reasons I want to participate in the mentorship program as well, and see what it can offer [Mentee 06 pre-program].
**Leadership Domain**	Teamwork	Increasing staff awareness about the NP role	I think lack of knowledge about the role is going to be another barrier, but my plan is to basically just have really good communication with everyone involved with cancer services. And also, other healthcare professionals in the Emergency Department and the regional hospitals, just to promote the role, and what I'm going to be doing, and just maintain good contact – communication [Mentee 08 pre-program].
	Confidence	Improving confidence	To me, it's really important that if I pick up the phone and call a doctor and say, "I'm concerned." That they have the faith to know that I'm not just panicking. I've done an assessment and my assessment might be wrong, but is most likely going to be accurate [Mentee 10 pre-program].

	Networking	Developing professional networks	And I just feel that because we're such a big regional area..., servicing so many patients, that it's good to create that network with other nurse practitioners. [Mentee 03 pre-program].
	Supporting health system	Promoting the service	The only thing I'm thinking is like what are my days going to look like and what equipment do I need to get the patient to contact me and like clinic space, where can I see the patient and how do I make the workflow works and how do I incorporate the team to look after the patient. And how to promote my service as well within the cancer centre [Mentee 10 pre-program].
