# Supplementary material for: Evaluation of the Cancer Nurses Society of Australia mentorship program for cancer nurse practitioners: brief research report
Source: Front Med (Lausanne). 2026 Jul 22;13:1753935. doi: 10.3389/fmed.2026.1753935 (PMC13449700; doi:10.3389/fmed.2026.1753935)
Supplement: Supplementary file 3 [file Data_Sheet_3.pdf]

Supplementary Table 3. Post-program interviews with mentees (N=10) and mentors (N=10). Summary of the domains, constructs and illustrative quotes

Domain	Construct	Program-specific construct	Examples of quotes
**Clinical Domain**	Clinical assessment	Assessing patients within the scope of practice/ assessment capability	Clinical [goal] was a bit more difficult because of physical assessments. It's hard to sort of do this way, but we would still go through them and talk about them and [my Mentor] would give me an advice. I found that really valuable because if you don't have an NP around you, you kind of expected to do that medical model of assessment but haven't been trained that way, so that was really good. I talked to him [my Mentor] mostly about my physical assessments [Mentee 02 post-program].<br><br>You know, but her [my Mentee] moving towards being a little bit more independent in her practice… I guess one of the things I sort of tried to talk to her about is, you know, you have the training, you have the skills. You can assess these patients and you can make a recommendation, and then present that recommendation to a doctor if you need to [Mentor 02 post-program].
	Clinical case-presentation	Case studies and case management	And the program provided exactly what I thought it would. I found it really beneficial. Our catch ups were good. I do case studies. I tell him [my mentor] what was happening at work. So, it was a really nice debriefing… Yeah, it was really just to make sure I was on track and then the other thing we would do case studies so I present a patient that I've seen and what my plan and outcome and things were and that was really beneficial as well because then he would provide feedback and make me think of it a little bit differently [Mentee 02 post-program].<br><br>We've done some case presentations, lots of pharmacological presentations and discussions but otherwise it [the program] did reach my expectations here [Mentee 03 post-program].
		Symptom management/side	Yeah, a lot of the topics that we did were looking at, cause we're seeing a lot more immunotherapies utilized in cancer care and because of that there's a broad range of toxicities that patients can get because of the immunotherapy treatment. So, a lot of that

		effects of cancer therapies	was sort of utilized in our time. So, obviously, really interesting cases that we had due to immunotherapy toxicity. So, [my Mentor], in particular, had some really interesting dermatological toxicity [knowledge] [Mentee 05 post-program].<br><br>Yes, we'd have discussions about pain management or nausea management or nutritional management or whatever but we would also have discussions for you know how would you make a change, how would you improve health services, not just for that individual, but for the next level. So, and I think that's what the mentorship relationship allows you to do is say well okay we fix it for this person, but how do we stop it happening or how do we change it for the future for other patients? [Mentor 06 post-program].
		Breaking bad news	I guess there was a case, we built a case study; and actually, there was a patient just at Christmas time, a woman who had had been in a remission of a lymphoma. So, working with a haematologist and just making sure that we, you know, we support the lady in the best way of breaking bad news, making sure that the, you know… the family members are extremely upset. Relax, and how could we have missed this and you know, and all that type of things. So, there's lots of discussions around that as well [Mentee 03 post-program].
	Diagnostic reasoning	Improving clinical diagnostic skills	So, we [My Mentee and I] talked a little bit about this and particularly focused on liver function tests (LFTs) because a lot of our drugs are hepatically metabolized and it can cause some derangement in LFTs. Sometimes those derangement in LFTs are acceptable; and you can continue on. Sometimes they require a dose reduction sometimes they require stopping up the treatment. So, she [my Mentee] did a literature search and gathered some evidence about common chemical therapies and, presented a case study to me and also to her group [Mentor 02 post-program].

	Clinical prescribing	Professional knowledge of pharmacotherapy and prescribing guidelines	Yeah, not difficulties, just I guess wariness and carefulness around prescribing and making sure that I'm checking everything. It's quite scary having to do that. It's one thing to do it as a student with your supervisor checking everything and whereas now being responsible for that having to make sure you've checked it against all the other medications that a patient's taking. It's definitely a slow process. And I think that'll continue for a while because, you know, you can never be too careful [Mentee 01 post-program].<br><br>I guess, she [my Mentee] was probably in the situation where she was seeing a lot of different cancers and a lot of different chemotherapy protocols and probably having built up that experience to know what is normal or abnormal and what is acceptable or unacceptable? So, I hope that exercise was good for her because it probably took a little bit back to pharmacology and pharmacodynamics and why these things happen to patients [Mentor 02 post-program].
		PBS and non-PBS prescribing	Yep, so there were lots of discussions around PBS and non-PBS prescribing. You know, what was appropriate, what wasn't appropriate in scope, how to extend, just go for practice to make sure that you were comfortable with regards to extending your scope to prescribe any medication that doesn't need to be on PBS looking at the PBS [Mentee 03 post-program].<br><br>So, yes, I guess that big learning curve from going through your training and then actually being legally able to prescribe that was, that was a big challenge for me starting as well, but it was, you know, it's really good to be throughout the mentoring program to be liaising and talking to [my Mentor] about the things we're prescribing, the different challenges with the PBS as well and knowing what's available to nurse practitioners that's actually on the PBS so that patients aren't paying significant amounts of money too because it was something that I hadn't even really considered, starting as a nurse practitioner.
		Prescribing cytotoxic medicines	Looking at, the extension of the cytotoxic therapy care plans while that in an EMR, there was lots of discussions about that [Mentee 03 post-program].

		Prescribing opioids and other drugs	I'm doing using a program which is probably the most difficult thing, but it's remembering like with those opioids and stuff how you have to write it out by hand and that type of thing. So, it's just getting advice on that [Mentee 04 post-program].<br><br>I would send her [my Mentee] information, if there was something I didn't know, I'd say, well, I'll have a look at my things at work and then I'd send her information or links or suggestions for education or I suppose one thing she asked when you said about education before, it was about prescribing medicinal cannabis and I prescribed that and then I found an education evening because she wanted to get more information about it because her patients always ask her about it. So, I put her on to the course you could do online [Mentor 07 post-program].
	Model of Care	Developing the model of care	Yeah, so [my Mentor] definitely helped with the model of care for me. And then especially with things like the rapid access clinic, I definitely utilized my mentor to get advice. I definitely sought his advice and got input from him with the more general running of clinics, but probably it was more about the actual rapid access clinic that I utilized him the most for just because of his experience in [other country]. He was actually involved in the development of the triaging tool that we utilized for our rapid access clinic here [Mentee 05 post-program].
		Improving the model of care	Yes, well, there was someone in a role that was very similar, and so, there was a model of care already in place, but I'm still currently developing all of that. So, it's only been sort of six or seven months now. So, still developing that model of care and the parameters around it [Mentee 01 post-program].<br><br>Yes, there's been things I've been bringing back to the group here and suggest if maybe we change. We can change the way we do things, but it didn't quite work out because it's to do with PBS prescribing and things like that with our electronic system. But he [my Mentor] shared his model of care with me. So that I could sort of have a look at how he works things there. But yeah, it was sort of was nothing new. It's just maybe adding to what the model of care is already [Mentee 02 post-program].

	Scope of practice	Defining the scope of practice within the transition period	When we started a mentorship program, it was good to be able to catch up with [my Mentor] when I needed to, as well as the monthly catch-up. So, we had those chats, a few themes I discussed with her regarding the conversations I've had with the doctors or how to get things through, how to set this up, how to do that, etc, which was really helpful. Particularly, when it comes to organizing my scope of practice and key position description and all that type of things, [my Mentor] was very helpful with all that [Mentee 04 post-program].<br><br>In my organization, some of colleagues that I'm aware of developed that process and I sent her my scope of practice, and the wording in my scope of practice, so that she could try and replicate that in her organization [Mentor 02 post-program].
		Expanding the scope of practice and related knowledge	I sat down and spoke to them about my scope of practice and about what I could do. As a nurse practitioner and then actually looked at sort of evolving my scope practice because as an as a nurse practitioner it's out of our scope to order CT scans and PET scans [Mentee 05 post-program].<br><br>She's [my Mentee] got a very good medical oncology colleague who's also supported it really well; and what was quite interesting that I was able to meet him and we were able to discuss, you know, where and when we were all working and how we were sort of expanding all of our scopes. I actually think my scopes expanded working with [my Mentee] over the last 12 months [Mentor 05 post-program].<br><br>I mean in certain areas like prescribing, clinical decision making, diagnostic reasoning and radiological interpretation, I would hope that we've done enough work together to expand all those areas of her scope [Mentor 05 post-program].

**Research Domain**	Research involvement	Not a priority/not in the role	Research. No, not really. No, nothing like that. Didn't really talk about research goals because I'm only just settling into this new role. Doing research isn't really something like that I'm looking at right now and, you know, I've mostly been focusing on how to get into grips with like the day-to-day expectations of my role clinically and just supporting myself like, you know, professionally with like education so that I'm competent in my role. So, and yeah, so no that isn't something that really came up [Mentee 08 post-program].<br><br>And in terms of research, you know, it's quite an early project for her [my mentee]. And so I was sharing the research program that I'm involved in [other state], and more of an insight at this point. I think the research aspect of her project is probably gonna come in the next couple of years [Mentor 05 post-program].
		Developing a research project	Research. We definitely talked about research at the last session because it's quite important because now that we are in a role that one is isolated; and we are the one that need to find evidence to support our service. So, we also talk about what kind of research to involve in, what should we do, how to do it, what was the experience in doing research. Yes, we talked about all of those definitely [Mentee 06 post-program].
**Education Domain**	Patient education	Improving patients' health literacy on medication management	So, we would discuss [with the mentee] the patient [case] and discuss how we would deliver that message to the patient. And I suppose the importance of that, you know, in terms of prescribing new, and then, you know, how to get the important messages across to the patient about that medicine [Mentor 05 post-program].<br><br>Yes, and I was trying to explain to nurses that we all provide education to patients, but it's a different level. So, you know, the radiation nurses, that the registered radiation nurse will provide the education about the treatment. But the nurse practitioner [takes it to] the next level that should also describe the side effects and how to manage those and also being that advanced practitioner she can then step in and manage those [Mentor 09 post-program].

	Staff education and mentorship	Staff clinical education and support	Working with the CNCs and the nursing staff in a therapy unit and also in terms of education opportunities, [My Mentor] was very, very kind in sharing her own stories of how to engage the nurses into education and use teachable moments for opportunities to educate and to promote and to highlight the importance of patient care [Mentee 10 post-program].<br><br>And then, education of staff. Well, I mean, [my mentee], she literally just recruited a team for her new project. So, I just helped here with a few resources that I've used for a symptom assessment tool that we developed in the UK. So, and we work through that tool and that resource to help her, educate her team [Mentor 05 post-program].
	Professional development/ career planning	Graduate education	And we talked about education. I'm actually studying at the moment. Yeah. I'm doing a Bachelor of Science in Psychology [Mentee 09 post-program].<br><br>She's [mentee] also taking on a psychology degree now, so she's hoping to encounter the psychology being part of her role as a nurse practitioner [Mentor 09 post-program].
		Professional development	Yeah, we talked about professional development; and I talked about like areas of my practice that I thought I needed improvement in and we discussed ways of doing that. So, we kind of discussed what sorts of courses might be useful or, you know, what reflections might be useful, and things like that [Mentee 01 post-program].
**Leadership Domain**	Teamwork	Increasing staff awareness about the NP role	From a nursing perspective, I think when I first started, it was about that awareness of nursing colleagues of what I could actually do… you know, because one day I was sitting in the office working with them as a CNC and the next day I'm in my own clinic working as an NP. So, I think as soon as we have those discussions; and they like it's got used to me being in a new role that then really helped with the flow of patient care [Mentee 05 post-program].

	Confidence	Improving confidence	The issues when you become endorsed as a nurse practitioner is almost that confidence to be able to independently do things. And I think [my mentor] gave me the confidence to be up, you know, be confident within myself. I think that was really useful just being able to run things by him and him going, yeah, I think, you dealt with that really well. So, I think just that reassurance. Yeah, it's more so just having the confidence with the assessments and things like that that [my mentor] reassured me with [Mentee 02 post-program].<br><br>I suppose we focused on leadership, but yeah, a lot of information about role definition and the confidence of a new nurse practitioner in this. We were able to reflect on my confidence and my leadership and you know to put [my mentee] at ease really that you know it's a constant learning experience; and just to reassure her really that you know every episode, whether it's me in, a patient or it's presented to a group of commissioners at hospital [Mentor 05 post-program].
	Networking	Developing and expanding professional networks	Sorry, when I registered for this program, I was about 18 months of being indoors. Because I don't have, there's no oncology nurse practitioners where I am. One of the first, there are other nurse practitioners in the organization. So, I just wanted to sort of have that support, make sure I was on the right track in my role and that sort of thing. And the program provided exactly what I thought. I found it really beneficial. [My mentor] was really good [Mentee 02 post-program].<br><br>Yeah, I recommended that she [my mentee] joined some, groups and get on board in some things and network and stuff. And again, part of her problem going to conferences was like a funding and you know, so we were talking about all those sorts of issues, but I did encourage her to get involved in some of CNSA groups because she was keen to look at [Mentor 04 post-program].

	Supporting health system	Expansion of service	Yeah, within the health service, I'm essentially the most senior nurse within cancer care services because of my role as the nurse practitioner. So often from an executive and leadership team they require advice on establishment or expansion of service. There's been a big focus, for example, within my health service on emergency avoidance and trying to keep our cancer care patients outside of emergency. So essentially in the last 9 months, I've established a rapid access clinic that's nurse practitioner led for people that have symptoms or side effects of their cancer and cancer treatment to come in for a review rather than present directly to emergency. And I found it really helpful to have, I guess, [my mentor’s] support during that because he was quite heavily involved over in [other country] with establishment of a similar service [Mentee 05 post-program].
